# Supplementary material for: Are carbon emissions trading and green financial instruments synergistic? -Comprehensive quantitative research based on content analysis
Source: PLoS One. 2024 Mar 7;19(3):e0298601. doi: 10.1371/journal.pone.0298601 (PMC10919601; doi:10.1371/journal.pone.0298601)
Supplement: S1 Data — (ZIP) [file pone.0298601.s001.zip › Data/地级市Z_F工作报告.zip!/┴╔─■/║∙┬1⁄2╡║╩╨/└┤╫╘╣1⁄2╓┌║┼í╛┬φ┐╦╩2╛▌═°í┐_2012.docx]

各位代表：

现在，我代表市人民政府向大会报告工作，请予审议，并请市政协各位委员和列席会议的同志提出意见。

一、2011年工作回顾

过去的一年，是我市经济社会发展的又一个丰收年。在市委的正确领导下，全市人民解放思想，开拓创新，真抓实干，圆满完成市四届人大二次会议确定的各项任务，开创了加快发展的崭新局面。

预计2011年，全市地区生产总值650亿元，增长15%；财政一般预算收入70.3亿元，增长25.7%；固定资产投资460亿元，增长35%；社会消费品零售总额274亿元，增长17.5%；实际利用外资4.4亿美元，增长100%；引进省外内资170亿元，增长35%；进出口总额13亿美元，其中出口8亿美元，增长23%；城镇居民人均可支配收入20150元，增长16%；农民人均纯收入7900元，增长20%。在国际国内经济环境不利的条件下实现较高增速，表明我市“十二五”开局良好，沿海经济强市建设强力推进！

一年来，我们主要在以下10个方面取得显著业绩：

第一，园区建设蓬勃发展。园区基础设施投入60.3亿元，增长25.4%，其中争取上级资金3.6亿元；累计投入178亿元。当年新引进项目280个，总投资1420亿元；新开工项目184个，总投资897亿元；在建项目246个，总投资975亿元；建成项目62个，总投资76亿元。园区总产值191.3亿元，增长142.8%；财政一般预算收入9.9亿元，增长65%。园区效益大幅跃升。

龙湾CBD基础设施配套齐备，海洋生态型中央商务区正在崛起。前所现代生态农业园区是辽宁沿海经济带唯一的农业园区；绥中滨海经济区被授予国家火炬计划数字技术特色产业基地和省高新技术产业开发区，兴城临海产业区被评为省新型工业化产业示范基地，打渔山泵阀产业园区成为省泵阀产业集聚区，建昌食品产业园区成为省农业特色产业集聚区，高新区创业中心被认定为国家级科技企业孵化器。园区建设跨入了历史最好时期！

第二，项目建设快速推进。项目年活动成果丰硕。投资5000万元以上在建项目489个，总投资超过3000亿元，项目数量和投资规模均列全省第8位。其中，20个投资10亿元以上项目进入“省重点工业项目”计划。柳条沟港区3个7万吨级泊位、渤海造船等71个重大项目竣工投产。投资130亿元的工业铝型材、投资50亿元的美玲工业园等216个重大项目开工建设；青山水库主体工程和移民动迁超额完成年度任务。猴山水库、石河港、大唐热电、15万吨TDI和南电三期等项目前期工作取得积极进展。

聚氨酯、泳装、有色金属深加工、海洋工程装备产业集群列入省重点。渤船重工销售收入超100亿元，中航黎明锦化机、葫芦岛钢管销售收入超10亿元。方大化工扭亏为盈，“锦化氯碱”成功复盘。淘汰落后产能企业7家，单位工业增加值能耗降低6%。民营企业增加值实现290亿元，增长22.9%；实现税收26亿元，增长28.7%；销售收入过亿元民营企业达到60家。项目支撑战略迸发出前所未有的强劲动力！

第三，县域经济稳步提升。新一轮县域经济“三年倍增”计划开局良好。预计县域地区生产总值、财政一般预算收入分别增长 27.2%和41.6%。粮食产量达到136万吨，创历史新高。水果、蔬菜、肉蛋奶、水产品产量分别达到102万吨、308万吨、73.2万吨、45万吨，分别增长12%、10%、10.4%和10%。

农业产业化晋档升级。新增设施蔬菜面积30万亩，达到110万亩。引进10亿元以上农产品加工项目10个，其中6个已开工。新建国家级粮油高产示范区20个、畜禽标准化养殖小区80个，新增地理标志商标3个，认证无公害农产品35个、绿色食品18个。新增农民专业合作社290家、农业产业化龙头企业 50家。完成农田水利骨干工程779项，实施节水滴灌工程13.5万亩，增殖放流对虾等3.5亿尾。兴城优质花生产业基地成为国家富民强县项目，连山塔山乡西堡村获全国一村一品示范村称号。一年来，县域经济保持倍增态势，新农村建设成果喜人！

第四，城区经济活力倍增。预计城区生产总值375亿元，财政一般预算收入38.4亿元，城区经济“三年倍增”任务圆满完成。进一步简政放权，向城区下放43项经济管理权限。行政区划调整取得重大突破，高桥镇平稳划至南票区，其他5个乡镇划转工作有序推进，南票百年矿区迎来近海发展的新机遇。

服务业全面发展。龙港商贸流通集聚区被评为首批省级服务业集聚区。富尔沃、大润发开业运营，恒大、首创、佳兆业等10个地产品牌企业落户。房地产施工面积873万平方米，增长63%；新开工面积400万平方米，增长43%。“万村千乡”市场工程覆盖全市99%的乡镇和98%的村，163万农民受益。现代服务业日益繁荣，股权基金合作进一步深化，与中国太平洋建设集团签订BT合作协议，第一批城投债券获批。全市金融机构贷款余额565.3亿元，比年初增长12.2%；新增贷款61.5亿元，比去年同期增长14%。

第五，对外开放成果显著。坚持外资盯住日韩港台、内资盯住东南沿海，全方位、多层次招商。推介项目490个，引进387个，落地270个。开创金融招商先例，与日本住友、韩国韩亚、香港亚联财等6家海外金融机构开展金融合作。温泉开发、城市综合体、新城新市镇等主题招商成效明显。海外并购项目6个，并购额4240万美元。对外劳务合作规模扩大，全年劳务输出2万人次。葫芦岛港实现外贸货物运输零的突破。

以节会促开放。成功举办首届国际泳装文化节，23个国家和地区700多名客商踊跃参加，签订贸易协议24个，总额1.7亿美元；签订投资协议23个，总额203亿元。成功举办泵阀产品博览会，参会企业313家，购销额5亿元；与20家企业签订投资协议107.2亿元。开创了我市走向世界的新局面！

第六，文化旅游新型支柱产业迅速兴起。规划了战国、秦汉、长城、古城等10大文化旅游板块。市本级和绥中、兴城文物局成立。姜女石遗址、兴城古城、圣水寺和长城列为“十二五”国家文化遗产保护项目，占全省总数的三分之一。《兴城古城保护规划》获国家批复，建昌东大杖子战国墓葬群入选第七批国家级文物保护单位，葫芦山庄成为全省唯一的五星级休闲农业与乡村旅游示范区。

文化品牌不断丰富。成功举办世界泳装设计大赛、模特大赛；“万人比基尼”群众性活动刷新吉尼斯世界纪录；“中华太极操”逐步推广，“中国太极岛”申报待批。群众文化节、读书节、文化大讲堂和文化广场活动丰富多彩。全年接待国内外游客1584万人次，增长21.2%；旅游总收入144.5亿元，增长56.7%。文化旅游产业正在向新型支柱产业大步迈进！

第七，城市建设步伐加快。城市功能进一步完善。龙程街、锦葫路等城市路网改造工程建成通车，高速公路葫芦岛东出口岛东线改造工程竣工，达到一级路标准，葫六线、龙湾大街南延改造启动实施。“901”人防工程建成使用，龙湾海滨人防工程主体完工。CBD“一场五馆”主体工程全部完工，文化馆投入使用，标志着我市即将结束没有现代化大型文体设施的历史。

以幸福和宜居为目标，开展了“我们的城市，我们的家园”城市环境综合整治活动，创新6项城市管理制度，实施14项提升工程，建成龙湾大街中段、海辰路、海滨南路3条鲜花大道，修建8处城市喷泉，城市基础设施实现填平补齐、优化升级，形成了人人维护环境、人人关爱家园的社会氛围。推进辽西绿色生态长廊建设，植树造林80.6万亩，森林覆盖率达到33.7%，荣获省绿化造林先进市三连冠。白狼山自然保护区晋升国家级保护区。近岸海域水质达标率100%，城市空气质量优良天数357天，占全年的97.8%。人居环境更加舒适优美。

第八，民生工程优质高效。上一年市政府的承诺全部兑现，我市社会保障指数在2011全国城市发展蓝皮书中排第8位。一是城镇实名制就业6万人，城镇登记失业率控制在3.7%以内。二是覆盖城乡的养老保险制度基本建立，27.4万城乡居民享受基础养老金待遇，11.5万企业离退休职工养老金人均每月上调136元。三是17万城乡低保对象实现应保尽保，低保救助标准再次提高。四是新农合参合率达到95%，基金使用率达到85%。五是农村中小学公用经费标准再次提高，低保家庭大学生救助实现全覆盖。六是城区供水管网改扩建工程提前一年完成，老城区实现24小时供水。七是建立大公交体系。重组市公汽公司，新增4条公交线路、1条滨海公路旅游线路，新增200辆公交车、200辆出租车，市民出行更加便捷；成功取缔城区营运三轮车，交通安全隐患得到有效治理。八是完成农村饮水安全工程314项，解决了13万农民饮水安全问题。九是劈山填海实施全省最大国有工矿棚户区改造项目，近10万户居民即将告别脏乱差的居住环境。十是完成扶贫攻坚任务，79个整村、9.4万贫困人口人均纯收入达到2116元，超过扶贫标准316元，实现稳定脱贫！

第九，各项社会事业繁荣发展。科教兴市战略深入实施，与清华大学共建社会实践基地，引进海外研发团队2个，兴城、绥中、建昌、龙港成为全国科普示范县，渤船重工成为国家级高新技术企业。推进教育均衡发展，改造学校97所，建成九年一贯制学校72所，高考录取率88.2%，职业教育毕业生就业率超过80%。文化体育事业健康发展，新建乡镇文化站46个，实现一村一个农家书屋。“迎全运、爱家乡、建辽宁”全民健身活动有声有色，我市在国际国内体育赛事中获金牌43枚，辽宁觉华岛围棋队获全国围甲联赛冠军。医药卫生体制改革不断深入，83家基层医疗卫生机构实施基本药物制度，基本药物配备和使用率均达到100%。基层医疗卫生单位综合改革通过省验收。人口出生率控制在6.58‰。

国防动员、军民共建、民兵预备役等工作成绩显著，荣获省级双拥模范城四连冠。我市成为全国民族团结进步教育基地。食品药品监管体系逐步健全，保障了人民群众饮食用药安全。物价保持基本稳定。国家安全、民族宗教、新闻出版、妇女儿童和防灾减灾工作不断加强，监察、审计、统计、质监、侨务、档案、气象、老龄、电力保障、对口支援、慈善、残联和红十字会等工作取得新成绩。

第十，民主法制建设得到加强。自觉接受人大的法律监督和工作监督、政协的民主监督，积极发挥各民主党派、工商联、无党派人士和人民团体参政议政作用。

办理人大代表建议73件、政协提案465件，办复率、见面沟通率为100%，满意和基本满意率为99.5%，落实和基本落实率达到64.1%，比上年提高2.6个百分点。坚持依法行政和重大问题公示、听证制度，推进政府决策科学化、民主化、法制化。狠抓纠风专项治理，提高行政效能，取消行政审批项目29项，公共服务水平再度提升。深入开展社会主义核心价值观教育，文明城市、文明社区和文明村镇创建活动取得成效。积极落实领导包案，稳妥处理信访问题。严厉打击各种违法犯罪活动，“清网行动”在全国现场会上介绍经验；安全生产形势总体稳定，突发事件处置能力得到提高，荣获省社会治安综合治理先进市五连冠、安全生产先进市三连冠，人民群众安全感明显增强！

各位代表，过去一年取得的成绩，是市委正确领导、科学决策的结果，是市人大和市政协有效监督、鼎力支持的结果，是历届班子打下坚实基础、持续发展的结果，是各地区各部门担纲履职、攻坚克难的结果，是全市人民和衷共济、团结奋进的结果。在此，我代表市人民政府，向各位人大代表、政协委员、各民主党派、工商联、各界人士和海内外朋友，向各驻葫单位、驻军部队、武警官兵、公安干警，向全市人民，致以崇高的敬意和衷心的感谢！

同时，我们也清醒地看到，对照建设沿海经济强市的目标，对照全市人民的期盼，政府工作还存在不少困难和问题。主要表现在：一是经济总量不大。地区生产总值仅占全省3%，财政一般预算收入仅占全省2.7%，赶超发展的基数较小。二是结构调整任务艰巨。高新技术产业比重较低，现代服务业相对滞后，民营经济发展不快。三是自我发展的内生动力不足。特别是牵动性大项目少，产业集群没有重大突破，难以支撑经济提速发展。由于形势变化和产业政策调整，徐大堡核电、千万吨炼油项目还在核准之中。四是民生保障水平有待提升。居民收入水平还不高，就业、就医、就学和居住、出行、安全状况需要进一步改善。五是发展环境还有差距。一些单位和部门服务意识不强，业务水平不高，工作效率较低，制约了沿海优势的发挥。对这些问题，我们一定高度重视，认真解决。

二、2012年工作安排

新的一年，面临新的挑战，蕴藏新的机遇。站在新的起点上，我们必须更加自觉地转变方式、加快发展，更加有效地挖掘潜力、发挥优势，更加负责地保障和改善民生、维护社会和谐稳定。

今年政府工作的指导思想是：坚持以科学发展观为统领，深入贯彻党的十七届六中全会、中央经济工作会议、省第十一次党代会和市第四次党代会精神，紧紧围绕保持经济平稳较快增长的中心，深刻把握“稳中求进，稳中求快，好中求快”的总基调，以建设沿海经济强市为战略目标，以转变经济发展方式和创新社会管理为主线，以推动文化改革繁荣为兴市之魂，以推进沿海经济带、县域经济、城区经济、民营经济和宜居滨城五大建设为重点，大力实施工业强市、项目支撑、沿海开放和民生保障四大战略，着力打造文化旅游新型支柱产业，努力为人民群众创造富庶文明幸福的新生活。

主要预期目标是：地区生产总值增长13%，财政一般预算收入增长15%，固定资产投资增长26%，社会消费品零售总额增长18%，实际利用外资增长40%，引进省外内资增长30%，出口总额增长20%，城镇居民人均可支配收入增长16%，农民人均纯收入增长16%。

为实现上述目标，重点抓好以下10个方面工作：

1、大力推进招商引资，提升沿海开发开放水平

实施重大项目招商攻坚计划。以世界500强、国内500强和行业100强为重点，实行一对一招商、叩门招商、驻点招商。每月都有主题招商活动，加大招商密度，提升招商质量。对10亿元以上重大项目实行专职专人跟踪对接服务，年内力争2～3户大企业落户。全市实际利用外资达到6.2亿美元，引进省外内资达到221亿元。

推进园区自主发展。优化园区管理体制，创新运行机制，让园区放开手脚、自主发展、快步前进。进一步完善园区基础设施，加快推进“七通一平”和标准化厂房建设，搞好研发、检测、营销、融资、物流等公共服务平台。加强园区生产要素保障，建设用地向园区倾斜，融资规模向园区集聚。强力推进项目入驻、投产，全市园区新引进项目要达到300个，新开工项目200个以上；每个省级重点园区力争新上投资5亿元以上项目10个，投资10亿元以上项目至少1个。

2、大力推进工业化建设，加大产业结构调整力度

突出重大项目建设。推进投资亿元以上重点项目363个，总投资3454亿元。其中，投资5亿元以上项目126个。加速推进投资130亿元的工业铝型材项目；开工建设投资69亿元的甲醇制烯烃及延伸加工、投资64亿元的路易LED、投资39亿元的名洋海洋工程、投资28亿元的中航黎明机械装备制造、投资21亿元的电网建设及改造等重大项目。

加速产业集群发展。推进聚氨酯、数字技术、泵阀、泳装、有色金属深加工、海洋工程装备等6个产业集群建设，每个产业集群年内至少引进1个投资超10亿元的项目，至少新增1个产值超10亿元的企业；产业集群总产值增长100%。积极发展高新技术产业，开发高技术含量、高附加值产品。规模以上工业增加值增长16%，单位工业增加值能耗下降5%。

3、大力推进城乡统筹发展，实现县域和城区经济整体跃升

加快县域经济产业化、工业化、城镇化进程。建设现代农业、高效农业，创建粮油高产示范区20个，改建、新建20万亩设施农业，新建畜禽标准化养殖小区74个、现代海洋牧场2万亩，建设20个市级新农村示范村。推进县域工业园区建设，6个10亿元以上农产品加工项目建成投产；兴城要在发展泳装产业的基础上，确立新的工业产业集群；绥中力争年内引进50家数字技术企业；建昌要规划建设北方冶金制造基地。推进新城新市镇建设，加快中心城镇建设步伐。按照30～50万人口标准，逐步完善县城功能、扩大县城规模。加大对建昌县的扶贫帮困力度，支持绥中县扩权改革，实现在全省位次前移。

启动新一轮城区经济“三年倍增”计划。按照城区享有县级权限的标准，进一步推进经济管理权力下放。加快服务业发展，传统服务业与现代服务业并重、生产性服务业与生活性服务业并举，按照销售收入50亿元以上的标准，推进24个服务业集聚区发展。恒大、新奥、首开等7个城市综合体项目加速推进，2个五星级酒店投入使用，1个五星级酒店主体完工。完成CBD扩区，建设商务金融服务中心。加快连山、龙港、兴城一体化建设步伐，统一规划、有序开发、共同发展。全面完成行政区划调整，启动南票新区建设，完成南票国有工矿棚户区改造，让6万多名工矿棚户区居民走出大山、喜迁新居！

4、大力推进民营经济提速，培育新的经济增长点

实施民营经济腾飞计划。鼓励全民创业、全民办实业，形成中小企业、微型企业发展浪潮。支持民间资本进入国家准许的所有领域，引导民企与国企对接合作，实现民企上市零的突破，完成海外并购项目3个。推进企业自主品牌建设，争创中国驰名商标2个、省级著名商标10个。壮大民营企业规模，销售收入超500万元的民营企业达到680家，民营经济增加值增长16%。

完善中小企业服务体系。加快小额贷款公司发展，建立中小企业信用担保机构风险补偿金制度，积极引进股权投资基金。推进县级创业辅导基地建设，设立中小企业发展专项资金，奖励获得驰名、著名商标、名牌产品和市长质量奖的民营企业，提升中小企业品质。制定扶持政策，全力支持成长型中小企业加快发展！

5、大力推进文化旅游产业发展，促进文化建设水平强势攀升

加快文化旅游产业发展。加强文化遗产保护，推进兴城古城保护和利用，制定姜女石、圣水寺整体保护规划。规划建设东大杖子战国墓葬群遗址公园。加快开发10大文化旅游板块，率先推出长城、温泉、红色文化和乡村旅游4个示范板块。建设沿海栈道，打造龙港、兴城、绥中“黄金海岸”。推动觉华岛国际旅游岛开发进程。办好第二届国际泳装文化节、泵阀博览会，发展节会经济。鼓励创办文化企业，培育文化创意产业，开工建设中国北方影视产业基地。

积极发展公共文化事业。完善市、县、乡、村公共文化服务设施功能，启动广播电视“户户通”工程，满足群众文化需求。开展优秀文学、艺术作品评选活动，促进群众性文化活动繁荣发展。建成CBD“一场五馆”，全部向市民开放。规划建设历史文化主题雕塑公园、国际和平广场、国际比基尼广场，展示文化旅游名城魅力。

6、大力推进基础设施和生态建设，提升生态宜居城市品位

启动新一轮城市总体规划修编，深化“我们的城市，我们的家园”活动。实施畅通工程，葫六线、龙湾大街南延路改造工程竣工，建兴高速、绕城公路、新老城区快速干道等12项道路工程开工。实施水源工程，青山水库主体完工，同步建设青山水库配套净水厂，开工建设猴山水库，新建农村饮水安全工程320处。实施港口工程，绥中港区通用码头主体竣工，柳条沟港区4个10万吨级泊位、兴城港区“一通一客”泊位开工建设，海铁联运实现重大突破，全面提升港口竞争力。实施职教工程，加速渤海船院迁址新建，建设全国高职现代教育体系示范区和中职教育示范园区。实施环保工程，严格环境影响评价，加强污染治理，新建5个垃圾处理场、2个污水处理厂，实施茨山河上游和五里河下游综合治理，力争年内实现流域水质达标。实施生态工程，启动国家森林城市创建工作，完成辽西绿色生态长廊建设任务，推进青山工程、凌河保护区工程建设，森林覆盖率达到36%；加强海岸线、湿地、滩涂和农村生态保护。努力打造四通八达、山青水秀、绿树成荫、卫生整洁、空气清新、美丽宜居的花园式生态宜居滨城！

7、大力推进民生工程，增强人民群众幸福感

2012年，市政府将集中力量为人民群众办好10件实事。一是实施新一轮扶贫攻坚，确保12.7万农村低收入群体人均纯收入达到2300元以上。二是实施农村医疗救助“一站式”结算服务，进一步提高农村低保和五保供养标准。三是每个乡镇建设1所标准化中心幼儿园，引导幼儿园连锁发展。四是实现城镇基本医疗保险门诊统筹制度全覆盖，全面解决43.7万无医保个人账户参保人员门诊待遇问题；实现政府办基层医疗卫生机构药品零差价销售，进一步解决看病贵问题。五是加强物业管理，逐步解决小区物业弃管问题。六是完善供暖设施，逐步取消小锅炉，实行集中供热，消除供暖低温区；改造下水管网设施，实现城区排水畅通。七是启动岔沟等18个城中村改造项目，推进老街老巷改造，提升老城区临街建筑档次；推进城区主要街路亮化升级，实现亮化向小街小巷和城郊延伸。八是国有工矿棚户区改造工程全面完工，保证入住，改善城乡居住条件。九是落实老年人保障政策，推进农村中心敬老院规范化管理，发展老年事业，让老年人老有所养、老有所乐。十是严格校车安全服务管理，切实保障学生安全，让家长放心！

同时，我们还要根据实际需求，为群众办更多实事，努力实现群众幸福指数与经济指标同步增长。

8、大力推进民主法制和精神文明建设，积极推动社会管理创新

认真执行人大及其常委会的决议决定，坚持报告工作制度；自觉接受人大的依法监督，虚心接受政协的民主监督，广泛听取各民主党派、工商联、无党派人士的意见和建议，主动接受人民群众的社会监督和新闻媒体的舆论监督。支持“两院”依法履职，发挥群团组织作用。加强基层政权建设，建立村委会主任培训机制，提升基层干部工作能力。重视群众来信来访，坚持标本兼治，着力解决引发不稳定因素的热点难点问题。推进“六五”普法，加强社会管理创新，强化社会治安综合治理，狠抓安全生产，严格食品药品监管，完善学校和幼儿园安全管理体系。全面推进应急管理体系建设，提高处置突发事件能力。以构建社会主义核心价值体系为重点，深入开展群众性精神文明创建活动，把学雷锋志愿服务工作纳入国民经济和社会发展规划，培育开放、友爱、创新、争优的城市精神。坚决整治干扰发展的现象，严肃查处损害发展的行为，积极创造更加宽松的政策环境、更加公平的法制环境、更加温馨的服务环境、更加和谐的人文环境，努力打造和谐葫芦岛！

9、大力推进公共服务能力建设，繁荣各项社会事业

突出就业和社会保障。新增城镇实名制就业4.2万人，城镇登记失业率控制在3.8%以内，保持零就业家庭动态管理为零。全市企业养老、医疗、失业、工伤和生育保险参保人数分别达到46.6万人、81.5万人、25.1万人、29.1万人和22.1万人；城镇居民养老保险实现应保尽保，新农保参保率达到80%以上，新农合参合率稳定在94%以上。统筹发展各项社会事业。创新引进人才、培育人才机制，让优秀人才脱颖而出，推进人才兴市。加快科技成果转化，高新技术产品增加值增长20%。继续实施学前教育三年行动计划，均衡发展义务教育，加快普及高中教育，优化职业教育，提升高等教育。深化医药卫生体制改革，完善城乡医疗服务体系。发展群众体育运动，全力打造“中国太极岛”，做好迎全运的各项工作。加强人口和计生工作，推进免费孕前优生健康检查，人口出生率控制在6.8‰以内。加强国防教育，支持部队建设，巩固军政军民团结。积极做好民族宗教、外事侨务、稳控物价、防灾减灾、福利慈善等各项工作。

10、大力推进政府自身建设，切实提高执政能力

坚持把政府自身建设摆到更加突出的位置，适应形势，转变职能，强化素质，提高效率，对人民负责，受人民监督，让人民满意。

建设诚信政府。坚持依法行政，推进简政放权，扩大政务公开，实现阳光行政。正视问题，敢于担当，讲操守、重品行，体察民意，顺应民心，言必信，行必果，以实际行动取信于民。

建设廉洁政府。坚持从严治政，推进惩治和预防腐败“五大系统”建设，注重自省自律，严格审计监察，规范从政行为。勤俭节约，艰苦奋斗，压缩“三公”开支，把有限财力用于改善民生、促进发展。

建设高效政府。坚持求真务实，谋于细、管于精、作于实，果断决策，雷厉风行，扑下身子，苦干实干。加强政府公务人员管理，推行办事承诺制、限时办结制、首问负责制，严肃工作纪律，严格责任追究，坚决治懒治庸。

建设为民政府。坚持以人为本，情系百姓，把群众的安危冷暖时刻放在心上。坚持群众观点，尊重群众、贴近群众，解难题、办实事，惠民富民。倾尽全力，不辞万难，全心全意为人民群众谋幸福！

各位代表！新期待凝聚力量，新使命催人奋进。让我们在市委的坚强领导下，满怀激情，团结奋斗，励精图治，顽强拼搏，共同谱写沿海经济强市建设新篇章，努力创造滨城人民富庶文明幸福的新生活！
